# COVID-19 and chronic diabetes: the perfect storm for reactivation tuberculosis?: a case series

**DOI:** 10.1186/s13256-021-03193-7

**Published:** 2021-12-16

**Authors:** Genesis P. Aguillón-Durán, Ericka Prieto-Martínez, Doris Ayala, Juan García, John M. Thomas, Juan Ignacio García, Brandon Michael Henry, Jordi B. Torrelles, Joanne Turner, Eder Ledezma-Campos, Blanca I. Restrepo

**Affiliations:** 1 School of Public Health, University of Texas Health Science Center at Houston, Brownsville Campus, Brownsville, TX 78520 USA; 2 Secretaria de Salud de Tamaulipas, 88630 Reynosa, Tamaulipas Mexico; 3 Secretaria de Salud de Tamaulipas, 87000 Ciudad Victoria, Tamaulipas Mexico; 4 Department of Human Genetics, South Texas Diabetes and Obesity Institute, School of Medicine, University of Texas Rio Grande Valley, 1214 W Schunior, UTRGV-Edinburg Campus, Edinburg, TX 78541 USA; 5 Population Health Program and Host Pathogens Interactions Program, Texas Biomedical Research Institute, San Antonio, TX 78229 USA

**Keywords:** Tuberculosis, COVID-19, SARS-CoV-2, Diabetes mellitus, Type 2 diabetes, Diagnostic delays

## Abstract

**Background:**

The coronavirus disease 2019 pandemic is predicted to have a net negative effect on tuberculosis control, with an estimated excess of 6.3 million tuberculosis cases and 1.4 million deaths by 2025. Programmatic issues such as the lockdown of tuberculosis services affect all patients, while biosocial factors have a differential impact on an individual’s risk for tuberculosis or adverse tuberculosis outcomes.

**Case presentation:**

We report three Hispanic cases of incident tuberculosis (two males, 43 and 44 years old; one female, 49 years old) after resolution of coronavirus disease episodes. Coincidentally, all cases shared a common risk factor: a chronic history poorly controlled diabetes.

**Conclusions:**

Our findings alert to the threat posed by the synergy between coronavirus disease and diabetes, on tuberculosis reactivation. In medium- to high-risk settings for tuberculosis, we recommend implementation of routine screening for latent tuberculosis infection in these cases, and preventive tuberculosis treatment in those who are positive.

## Background

The coronavirus disease 2019 (COVID-19) pandemic is predicted to become a double-edged sword for tuberculosis (TB) control [1]. On the positive side, comprehensive preventive measures against COVID-19 such as self-quarantine, social distancing, and use of face masks is likely to reduce the spread of *Mycobacterium tuberculosis*. However, there is an anticipated net negative effect of COVID-19 on TB control, including an increase in the number of TB cases and deaths, and the proportion of drug-resistant TB [1]. Two types of interrelated factors can contribute: the first type are programmatic issues that affect all TB patients in a community. These include the lockdown of TB diagnosis and treatment services, and the redirection of the already limited resources for national TB programs worldwide to address the urgency of the COVID-19 pandemic. A model estimated that between 2020 and 2025 there will be a global excess of active TB cases (3.1–10.7%) and deaths (4–16%) as a result of the COVID-19 pandemic, setting back global TB control efforts by at least 8 years [2].

The second type are biological or social factors that can contribute to the hypothesized bidirectional synergy between severe acute respiratory syndrome coronavirus 2 (SARS-CoV-2) and *Mycobacterium tuberculosis* [3]. First, both microbes primarily target the lung. Second, both share social risk factors (for example poverty, overcrowding, malnutrition, and poor access to healthcare), which increase the risk of disease transmission, progression, and poor TB treatment outcomes [4–6]. Third, both diseases share biological risks, such as male gender or diabetes. Diabetes increases the risk of TB development or adverse treatment outcomes, and is also a risk factor for more severe COVID-19 presentation and increases the risk of death [4, 7–9]. Fourth, there is reported immunosuppression in COVID-19 patients that would be expected to favor *M. tuberculosis* growth. These include reduction in CD4 and CD8 counts, functional exhaustion of these T cells [10] and heightened production of interleukin (IL)-10 in response to the viral infection [11], and the widespread, often unregulated and inappropriate use of immunosuppressive treatments (for example steroids, tocilizumab) in many countries [12]. Thus, the convergence of these biosocial factors would be expected to increase the risk of adverse outcomes for COVID-19 or TB. However, clinical evidence is still limited, albeit beginning to show support. The first published cohort of COVID-19 and TB provides examples of TB preceding, overlapping, or following COVID-19 infection [13]. Some studies have reported more severe TB disease, delayed recovery, and higher death in patients with both TB and COVID-19 [3, 4, 13–15]. Likewise, TB prevalence was higher among patients with severe COVID-19, when compared with nonsevere cases [3]. Thus, the need for integrated care of both types of diseases and, hence, knowledge of the individual risk factors that retro-feed on each other.

However, to date, the role of COVID-19 in boosting TB development is yet to be established [3]. We describe our findings in three individuals with newly diagnosed TB after recovery from COVID-19 (Table 1). These cases alert to the heightened risk for reactivation TB in patients recovering from COVID-19 with a chronic history of poorly controlled diabetes.

**Table 1:** Characteristics of newly diagnosed tuberculosis patients with a multiyear history of diabetes and a recent history of COVID-19

	TR-241	TR-243	TR-247
Demographics^a^			
Age in years, Sex	43, male	44, male	49, female
COVID-19 history			
Timing prior to TB diagnosis	3 months	6 months	4 months
Anti-SARS-CoV-2 IgG at TB diagnosis^b^	Positive	Positive	Positive
Symptoms at the time of the COVID-19 episode:			
Cough	Yes	Yes	Yes
Fever, chills	Yes	Yes	Yes
Fatigue	Yes	Yes	Yes
Loss of smell and taste	Yes	Yes	Yes
Shortness of breath	Yes	No	No
Body aches	Yes	Yes	No
Duration of TB signs and symptoms prior to reporting to the TB clinic (days)			
Cough	90	180	120
Productive cough	60	90	60
Fever, chills	14	15	14
Chest pain	3	N/R	N/R
Weight loss	60	60	60
Fatigue	N/R	N/R	30
TB diagnosis			
Acid-fast bacilli smear grade (bacilli/field)	> 10	> 10	> 10
*M. tuberculosis* culture^c^	Positive	Positive	Positive
TB risk factors and other medical conditions			
Body mass index	13.4	23.7	29
BCG vaccine	Yes	Yes	Yes
HIV	Negative	Negative	Negative
Smoking	No	No	No
Alcohol excess and illicit drugs^d^	No	Yes	No
Type 2 diabetes	Yes	Yes	Yes
Years with type 2 diabetes	13 y	5 y	6 y
HbA1c	8.2%	7.5%	10.6%
Fasting blood glucose (mg/dl)	105	138	126
Diabetes medications^e^	Metformin	No	Glibenclamide metformin
Other medical conditions	Peripheral neuropathy	None	High blood pressure
History of past TB or TB exposure			
Self-reported past exposure to a TB patient	No	No	Yes, > 2 years ago
Prior testing for latent TB infection	No	No	No
Past history of active TB	No	No	No

*N/R* symptom not reported, *TB* tuberculosis, *SARS-CoV-2* SARS coronavirus 2, *COVID-19* coronavirus disease 2019, *HIV* Human immunodeficiency virus, *HbA1c* hemoglobin A1c

^a^All are Hispanic, White

^b^Anti-SARS-CoV-2 IgG assay, Abbott Laboratories, Abbott Park, IL

^c^*M. tuberculosis* spp. identified with BioLine MPT64 Rapid, Standard Diagnostics, Korea

^d^TR-243: > 10 drinks and cocaine on weekends

^e^TR-247: metformin 1700 mg/d; TR-249: Metformin 500 mg/d and glibenclamide 2.5 mg/day

## Case presentation

Two males (43 and 44 years old) and one female (49 years old) presented with signs and symptoms suggestive of active TB (Table 1) to the Centro Regional de Tuberculosis in Reynosa, Tamaulipas. They reported no pulmonary symptoms prior to the development of a COVID-19 episode 3–6 months ago. Their COVID-19 symptoms gradually disappeared, except for a persistent dry cough that gradually evolved into a productive cough (60 days ago for TR-241 and TR-247 and 90 days ago for TR-243), and was accompanied by dramatic weight loss and the reappearance of fever and chills 14–15 days prior to reporting to the TB clinic. The three cases were diagnosed with pulmonary TB supported by abnormal chest x-rays, positive acid-fast sputum smears (> 10 bacilli/field), and culture confirmation of *M. tuberculosis* spp. Their previous infection with SARS-CoV-2 was confirmed by positive anti-SARS-CoV-2 IgG titers. Informed consent was obtained from both participants as part of a parent study.

Besides COVID-19, other host factors or medical conditions influencing TB risk were examined (Table 1). A consistent finding was a chronic history of type 2 diabetes (≥ 5 years) with poor glucose control (HbA1c ≥ 7.5%). We cannot ascertain whether these were cases of primary progressive TB disease or of an incipient TB that was smoldering at the time of the COVID-19 diagnosis, or if this was a reactivation of a latent *M. tuberculosis* infection prompted by the COVID-19 episode. We consider the latter is more likely given that: (i) none of the cases reported pulmonary symptoms prior to the COVID-19 episode, (ii) two had no knowledge of previous exposure to a TB patient and one had a past exposure (Table 1), and (iii) there was a gradual appearance of a productive cough and the reemergence of fever and chills after the COVID-19 episode was resolved.

## Discussion

Given that active TB disease takes weeks or months to develop, and that we are at an initial stage of the COVID-19 pandemic, it is still early to evaluate the actual impact of COVID-19 on *M. tuberculosis* reactivation. Our findings alert us to be prepared in several ways.

First, diabetes and the SARS-CoV-2 infection are likely to retro feed each other to magnify the risk of *M. tuberculosis* reactivation to active TB. Diabetes is a major comorbidity for COVID-19 patients, resulting in poorer outcomes (RR 2.38, *p* < 0.001), death (RR 2.12), and more severe disease (RR 2.45) [16]. Diabetes is also a well-established risk factor for TB [17, 18], and for adverse TB disease outcomes [19, 20]. SARS-CoV-2 infection is associated with immunosuppression that persists past the COVID-19 episode, and likely synergizes with TB to favor primary or reactivation TB [21, 22]. Thus, we predict that the threefold higher risk of active TB development in diabetes patients is further amplified by SARS-CoV-2 infection.

Second, in our post-COVID-19 TB cases, the continuum of cough throughout both episodes masked the suspicion of an emerging TB and likely contributed to the significant delay in its diagnosis (60–90 days with productive cough) and high *M. tuberculosis* burden in their sputum (> 10 bacilli/field). TB diagnostic delays and high bacillary burden are associated with expanded community TB transmission and poorer TB prognosis [23], which is an added concern for post-COVID-19 TB cases.

We recommend clinicians and public health workers to regard COVID-19 patients with diabetes and latent *M. tuberculosis* infection as having a heightened risk for TB. In high incidence settings for latent *M. tuberculosis* infection, patients with COVID-19 and diabetes should be alerted and educated to “think TB” in their post-COVID-19 recovery period, to reduce patient and healthcare provider diagnostic delays. In low- to medium-risk settings for TB, screening for latent *M. tuberculosis* infection and treatment should be considered in diabetes patients recovering from COVID-19. Of note, current guidelines by the World Health Organization do not currently prioritize latent *M. tuberculosis* infection testing in diabetes patients [24]; however, this pre-COVID-19 recommendation should now be a consideration for those recovering from this viral infection. Finally, in developed countries where prophylactic TB treatment is administered, COVID-19 patients with diabetes comorbidity should be added to their targeted latent *M. tuberculosis* infection testing and treatment program.

## Conclusion

Our findings alert to the heightened risk of *M. tuberculosis* reactivation among COVID-19 patients with a history of chronic and poorly controlled diabetes. In light of our findings, further research is needed to elucidate the underlying mechanisms and identify those who may be at highest risk, for targeted monitoring and management. In the meantime, we suggest a framework for new guidelines to prevent active TB in individuals affected with the COVID-19 and diabetes.

## Data Availability

The datasets used and/or analyzed during the current study are available from the corresponding author on reasonable request.
